# The effect of pH on the aerobic and hypoxic cytotoxicity of SR4233 in HT-29 cells.

**DOI:** 10.1038/bjc.1993.409

**Published:** 1993-10

**Authors:** L. D. Skarsgard, M. W. Skwarchuk, A. Vinczan, D. J. Chaplin

**Affiliations:** Department of Medical Biophysics, BC Cancer Research Centre, Vancouver, Canada.

## Abstract

We have observed that low pH can substantially potentiate the cytotoxic effect of the bioreductive drug SR4233 in aerobic HT-29 human tumour cells. No such potentiation was observed under hypoxic conditions. This pH effect might be relevant both to the therapeutic effectiveness and to the normal tissue toxicity of this new agent.


Br. J. Cancer (1993), 68, 681–683 © Macmillan Press Ltd., 1993

SHORT COMMUNICATION

### The effect of pH on the aerobic and hypoxic cytotoxicity of SR4233 in HT-29 cells

L.D. Skarsgard[1], M.W. Skwarchuk[1], A. Vinczan[1] & D.J. Chaplin[1,2]

[1]*Department of Medical Biophysics, BC Cancer Research Centre, 601 West 10th Avenue, Vancouver, BC V5Z 1L3, Canada and* [2]*Vascular Targeting Group, CRC Gray Laboratory, Mount Vernon Hospital, Northwood, Middlesex HA6 2JR, UK.*

**Summary** We have observed that low pH can substantially potentiate the cytotoxic effect of the bioreductive drug SR4233 in *aerobic* HT-29 human tumour cells. No such potentiation was observed under hypoxic conditions. This pH effect might be relevant both to the therapeutic effectiveness and to the normal tissue toxicity of this new agent.

The bioreductive drug 1,2,4-Benzotriazin-3-amine 1,4-Dioxide (SR4233 or WIN59075) has been shown to have a remarkably selective toxicity for hypoxic cells (Zeman *et al*., 1986), a property which has stimulated much interest in its potential as an adjunct to radiation and chemotherapy. It is believed that the active form of the agent is an oxygen-sensitive, 1-electron reduction product (Laderoute *et al*., 1988; Baker *et al*., 1988). A potentially important secondary property of SR4233 is its reported ability to act as a radiosensitiser of aerobic cells which receive a pre- or post-irradiation exposure to the drug under hypoxic conditions (Zeman & Brown, 1989). These authors suggested that this characteristic might be effective in dealing with the problem of transient hypoxia known to occur in experimental tumours (Chaplin *et al*., 1986, 1987). It has also been shown that hydralazine can potentiate the cytotoxic effect of SR4233 in a mouse tumour system (Brown, 1987).

While there has been extensive study of the effect of hypoxia on the cytotoxicity of SR4233, there has been relatively little attention given to the influence of other microenvironmental factors which might be important to the anti-tumour potential of this drug (Tocher *et al*., 1990; Herman *et al*., 1990). In this paper we describe the influence of pH on the cytotoxic effect of SR4233 under both aerobic and hypoxic conditions.

### Materials and methods

The cytotoxicity of SR4233 was examined in human colon adenocarcinoma HT-29 cells (from American Type Culture Collection, Rockville, Maryland, USA). These cells were grown as monolayer cultures in McCoy's 5A medium, supplemented with 10% foetal bovine serum (both from Gibco, Burlington, Ontario, Canada), penicillin (80 units $ml^{-1}$) and streptomycin (80 $\mu g\ ml^{-1}$) and sodium bicarbonate (2.2 $g\ l^{-1}$). Monolayers were sub-cultured using a 0.25% trypsin solution. For experiments, cell suspensions were prepared at $2 \times 10^5$ cells $ml^{-1}$ in normal growth medium lacking sodium bicarbonate but supplemented with 20 mM BIS-TRIS (bis[2-Hydroxyethyl]imino-tris-hydroxymethyl] methane, from Sigma Chemical Co., St. Louis, MO, USA) for control of pH. Cell suspensions were prepared at each pH to be investigated, ranging from 6.0 to 7.4, using pH-adjusted medium. Nine ml of cell suspension were then placed in each 24 wide-mouth, 50 ml Erlenmeyer flasks. The vessels were fitted with stoppers which provided ports for gas in, gas out, and sampling. The flasks were placed in a 37°C rotary shaker bath and each flask was gassed with humidified air or $N_2$ (14 l $min^{-1}$) in a 37°C warm room. Following 45 min gassing, SR4233 aliquots were added to yield the prescribed experimental concentration. Controls and a single drug concentration (1000 μM for the aerobic and 40 μM for the hypoxic treatment) were used. After 60 min incubation time, aliquots were removed from each flask, spun down and washed twice to remove the drug. Appropriate inocula were plated in 60 mm petri dishes for colony formation measurement. The pH of the cell suspensions was checked at the end of the 60 min incubation period. Although the pH generally decreased during this period, the changes were small: suspensions with a nominal pH of 6.60, for example, were found to have final pH's of 6.52 to 6.60 at the end of the incubation period.

### *Drug preparation*

SR4233 was kindly provided by Dr M. Tracy of Stanford Research Institute, Menlo Park, CA, USA. Approximately 45 min before the SR4233 treatment was to begin, a fresh stock solution of SR4233 was prepared by dissolving the drug in 37°C medium (buffered by BIS-TRIS) for 10 min, followed by sonication at 37°C for 5 min. The concentration of SR4233 was 10 mM (solubility limit is 13.5 mM). The stock solution was sterile-filtered and diluted to provide appropriate concentrations.

### Results

Figure 1 shows the effect of pH on the cytotoxicity of SR4233 in HT-29 human colon adenocarcinoma cells, for both aerobic and hypoxic conditions. The cells were incubated at 37°C for 1 h in complete medium containing 1000 μM or 40 μM SR4233 for aerobic or hypoxic conditions, respectively. These conditions were chosen to yield approximately equivalent cell kill at pH 6.6 and they illustrate the striking potentiation of SR4233 cytotoxicity that occurs with hypoxic conditions. It can be seen that the plating efficiency of these cells, in the absence of drug, is unaffected by pH and is approximately the same under both aerobic and hypoxic conditions (range 50–86%). Under aerobic conditions, the cytotoxic effect of 1000 μM SR4233 is strongly potentiated by low pH; the cell kill at pH 6.0 is approximately 300-fold greater than at pH 7.4. Under hypoxic treatment, however, low pH seems to have, if anything, a slightly protective effect against SR4233 cytotoxicity.

### Discussion

Our studies with HT-29 human tumour cells have shown that the cytotoxic effect of SR4233 is greatly enhanced under

Correspondence: L.D. Skarsgard, PhD., Department of Medical Biophysics, BC Cancer Research Centre, 601 West 10th Avenue, Vancouver, BC V5Z 1L3, Canada.
Received 27 October 1992; and in revised form 25 May 1993.

**Figure 1** Effect of pH on the cytotoxicity of SR4233 in HT-29 human colon adenocarcinoma cells. Exposure for 1 h at 37°C and the indicated pH to SR4233 under aerobic conditions (open circles, [SR4233] = 1000 μM) or hypoxic conditions (closed circles, [SR4233] = 40 μM). The results are pooled from three different experiments.

hypoxic conditions, consistent with the results of Zeman *et al.* (1986) in several different rodent and human cell lines. The potentiation of the *aerobic* cytotoxicity of SR4233 which we found associated with low extracellular pH, has not been previously described, to our knowledge. Tocher *et al.* (1990) reported that acidic pH led to increased activity of reduced SR4233 in a φ × 174 DNA damage assay under hypoxia but no aerobic results were given. Herman *et al.* (1990) reported that low pH (6.45) enhanced the hypoxic cytotoxicity of SR4233 against a mouse tumour cell line, FSaIIC, by approximately 2-fold, but had no effect on the aerobic response; results with that cell line were unusual, however, in that hypoxia only enhance the cytotoxicity of SR4233 by a factor of 2 to 3.

If the acidic potentiation of the aerobic cytotoxicity of SR4233 reported here is found to be a general property of the drug (we have recently observed it as well in SiHa cells, a human cervical squamous carcinoma cell line), it adds an interesting third dimension to the potential therapeutic value of this drug, in addition to the hypoxic cytotoxicity and the aerobic radiosensitisation (Zeman *et al.*, 1986; Zeman & Brown, 1989). The reported aerobic radiosensitization has been identified as a potentially useful property of the drug in treating tumours where intermittent blood flow and its consequent transient hypoxia may compromise the hypoxic cytotoxicity of SR4233. The effect was credited with producing a significant therapeutic gain in fractionated irradiation of mouse tumours (Brown & Lemmon, 1991). Since low pH and hypoxia are conditions which will often coexist in certain tumour cell subpopulations and since acidic conditions are probably less variable and longer-persisting than (intermittent) hypoxia in solid tumours, the low pH potentiation of SR4233 cytotoxicity described here could also play a role in contributing to therapeutic gain.

It is also possible that the potentiation of SR4233 toxicity by acidic pH under aerobic conditions could contribute to the potentiation observed when SR4233 is combined with agents which induce ischemia (Sun & Brown, 1989; Edwards *et al.*, 1991; Braunschweiger *et al.*, 1991). It would be expected that agents such as FAA and TNF, which induce permanent vascular occlusion in a large proportion of tumour vessels, would result in a significant acidification of the tumour micromilieu. Indeed, since acidic metabolites will diffuse throughout the tumour mass until removed via the vascular system, significant pH reduction around and close to functioning vessels might be expected. Based on our results reported here, such an acidification would make these aerobic cells more susceptible to killing by SR4233.

We have not addressed the mechanism responsible for the preferential effect of acidic conditions on the aerobic cytotoxicity of SR4233. However, it has been suggested that the toxicity of SR4233 under aerobic conditions is due to the production of damaging oxygen species derived from the back oxidation of the one-electron reduction product (Laderoute *et al.*, 1988; Baker *et al.*, 1988). If this is true, acidic pH could increase aerobic toxicity by decreasing the removal or increasing the production of damaging oxygen species. Whatever the exact mechanism responsible, if the limiting normal tissue toxicity observed when clinical trials are initiated is related to the aerobic toxicity *in vitro*, tissue pH could play a role in determining the severity of toxicity.

In summary, the *in vitro* results demonstrate that the aerobic toxicity of SR4233 is enhanced under conditions of acidic pH. In contrast, no enhancement of the hypoxic toxicity of SR4233 by acidic pH could be demonstrated. This finding may indicate that pH could play a role in both the tumour cytotoxicity and normal tissue damage.

This work was supported by the B.C. Health Research Foundation, the National Cancer Institute of Canada and the B.C. Cancer Foundation.

### References

BAKER, M.A., ZEMAN, E.M., HIRST, V.K. & BROWN, J.M. (1988). Metabolism of SR4233 by Chinese hamster ovary cells: basis of selective hypoxic cytotoxicity. *Cancer Res.*, **48**, 5947–5952.

BRAUNSCHWEIGER, P.G., JONES, S.A., JOHNSON, C.S. & FURMANSKI, P. (1991). Interleukin-1α induced tumour pathophysiologies can be exploited with bioreductive alkylating agents. *Int. J. Radiat. Biol.*, **60**, 369–372.

BROWN, J.M. (1987). Exploitation of bioreductive agents with vasoactive drugs. In *Proceedings of the Eighth International Congress of Radiation Research, Volume 2*, Fielden, E.M., Fowler, J.F., Hendry, J.H. & Scott, D. (eds). pp. 719–724. Taylor & Francis, London.

BROWN, J.M. & LEMMON, M.J. (1991). Tumor hypoxia can be exploited to preferentially sensitize tumors to fractionated irradiation. *Int. J. Radiat. Oncol. Biol. Phys.*, **20**, 457–461.

CHAPLIN, D.J., DURAND, R.E. & OLIVE, P.L. (1986). Acute hypoxia in tumors: implications for modifiers of radiation effects. *Int. J. Radiat. Oncol. Biol. Phys.*, **12**, 1279–1282.

CHAPLIN, D.J., OLIVE, P.L. & DURAND, R.E. (1987). Intermittent blood flow in a murine tumor: radiobiological effects. *Cancer Res.*, **47**, 597–601.

EDWARDS, H.S., BREMNER, J.C.M. & STRATFORD, I.J. (1991). Induction of tumor hypoxia by FAA and TNF interaction with bioreductive drugs. *Int. J. Radiat. Biol.*, **60**, 373–377.

HERMAN, T.S., TEICHER, B.A. & COLEMAN, C.N. (1990). Interaction of SR-4233 with hyperthermia and radiation in the FSaIIC murine fibrosarcoma tumor system *in vitro* and *in vivo*. *Cancer Res.*, **50**, 5055–5059.

LADEROUTE, K., WARDMAN, P. & RAUTH, A.M. (1988). Molecular mechanisms for the hypoxia-dependent activation of 3-amino-1,2,4-benzotriazine-1, 4-dioxide (SR4233). *Biochem. Pharmacol.*, **37**, 1487–1495.

SUN, J.R. & BROWN, J.M. (1989). Enhancement of the antitumor effect of Flavone acetic acid by the bioreductive drug SR4233 in a murine carcinoma. *Cancer Res.*, **49**, 5664–5670.

TOCHER, J.H., VIRK, N.S. & EDWARDS, D.I. (1990). DNA damaging effects and voltammetric studies on the hypoxic cell toxin 3-amino-1,2,4-benzotriazine-1,4-dioxide, SR4233, as a function of pH. *Biochem. Pharmacol.*, **40**, 1405-1410.

ZEMAN, E.M., BROWN, J.M., LEMMON, M.J., HIRST, V.K. & LEE, W.W. (1986). SR4233: a new bioreductive agent with high selective toxicity for hypoxic mammalian cells. *Int. J. Radiat. Oncol. Biol. Phys.*, **12**, 1239-1242.

ZEMAN, E.M. & BROWN, J.M. (1989). Pre- and post-irradiation radiosensitization by SR4233. *Int. J. Radiat. Oncol. Biol. Phys.*, **16**, 967-971.


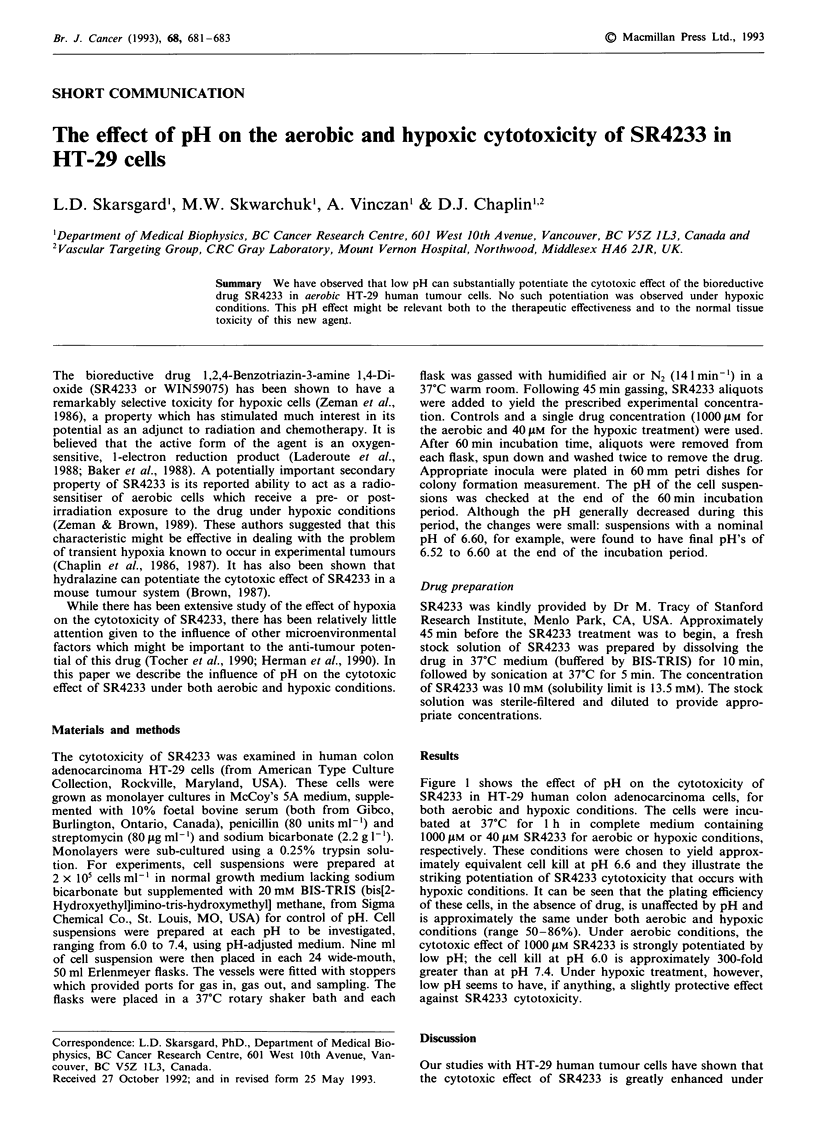

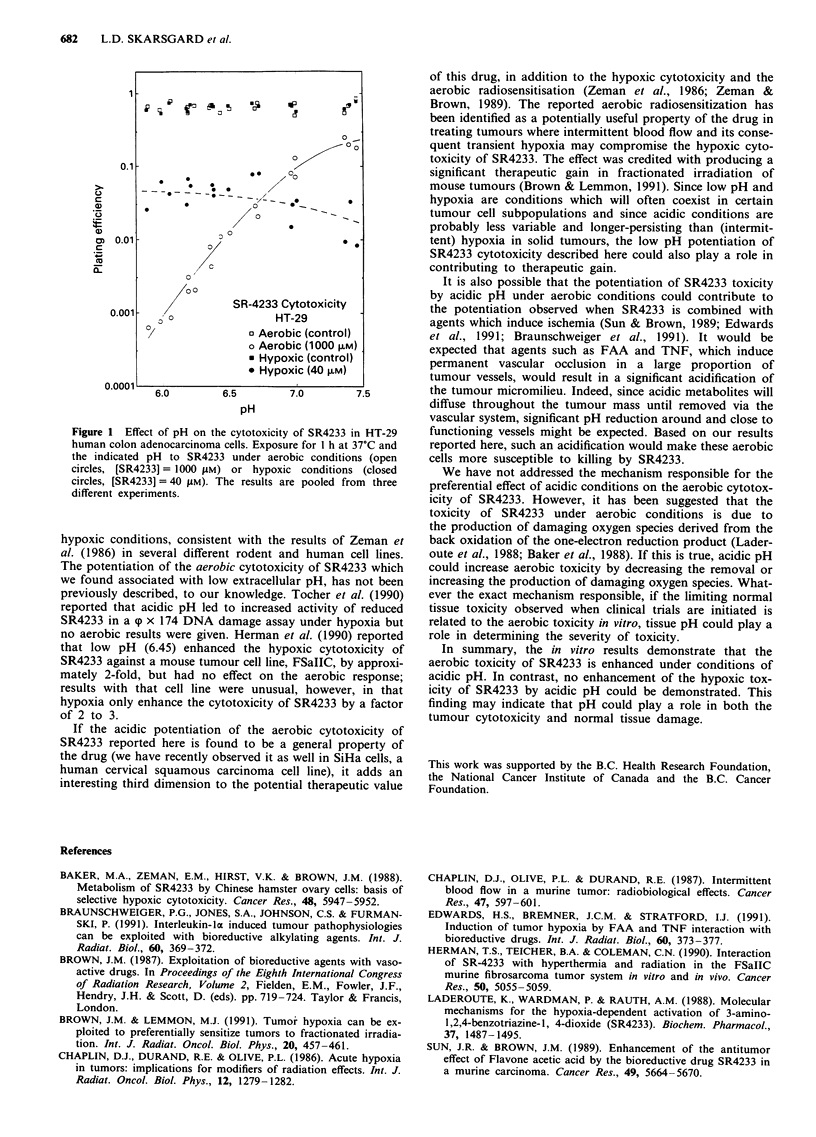

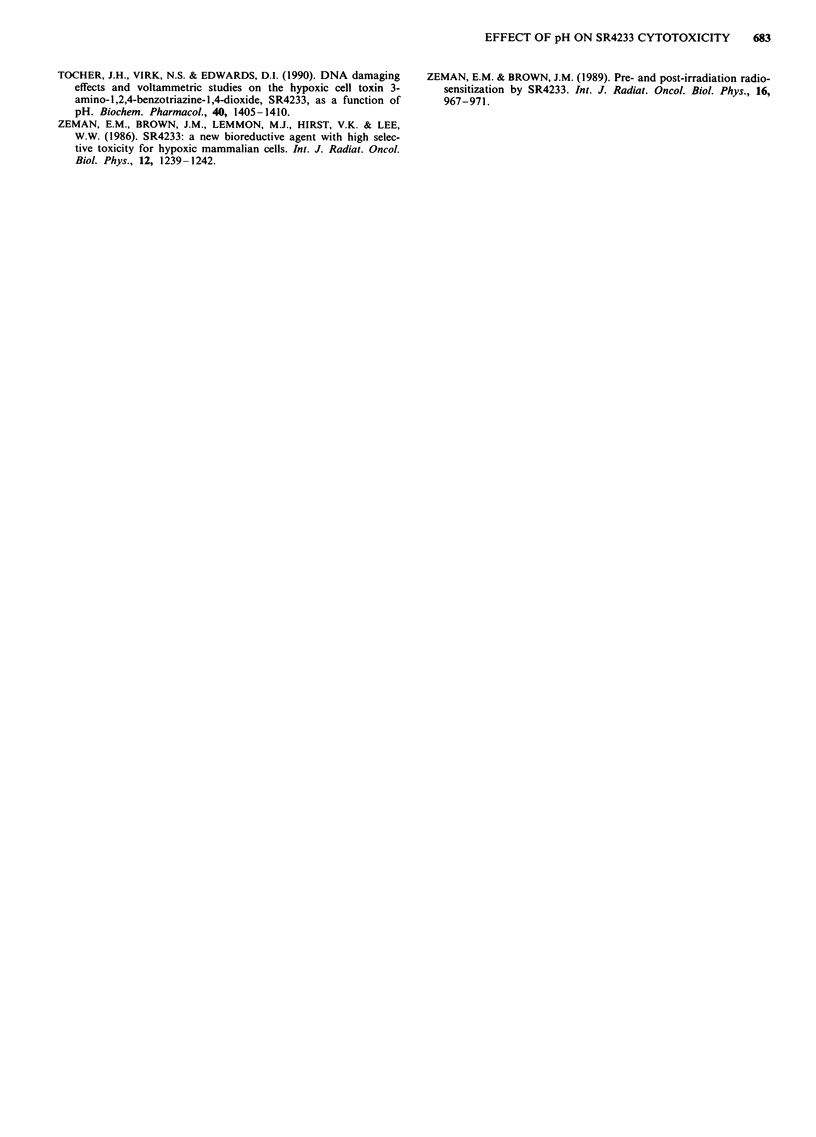


## References

[OCR_00251] Baker M. A., Zeman E. M., Hirst V. K., Brown J. M. (1988). Metabolism of SR 4233 by Chinese hamster ovary cells: basis of selective hypoxic cytotoxicity.. Cancer Res.

[OCR_00258] Braunschweiger P. G., Jones S. A., Johnson C. S., Furmanski P. (1991). Interleukin-1 alpha-induced tumour pathophysiologies can be exploited with bioreductive alkylating agents.. Int J Radiat Biol.

[OCR_00269] Brown J. M., Lemmon M. J. (1991). Tumor hypoxia can be exploited to preferentially sensitize tumors to fractionated irradiation.. Int J Radiat Oncol Biol Phys.

[OCR_00274] Chaplin D. J., Durand R. E., Olive P. L. (1986). Acute hypoxia in tumors: implications for modifiers of radiation effects.. Int J Radiat Oncol Biol Phys.

[OCR_00279] Chaplin D. J., Olive P. L., Durand R. E. (1987). Intermittent blood flow in a murine tumor: radiobiological effects.. Cancer Res.

[OCR_00284] Edwards H. S., Bremner J. C., Stratford I. J. (1991). Induction of tumour hypoxia by FAA and TNF: interaction with bioreductive drugs.. Int J Radiat Biol.

[OCR_00289] Herman T. S., Teicher B. A., Coleman C. N. (1990). Interaction of SR-4233 with hyperthermia and radiation in the FSaIIC murine fibrosarcoma tumor system in vitro and in vivo.. Cancer Res.

[OCR_00295] Laderoute K., Wardman P., Rauth A. M. (1988). Molecular mechanisms for the hypoxia-dependent activation of 3-amino-1,2,4-benzotriazine-1,4-dioxide (SR 4233).. Biochem Pharmacol.

[OCR_00301] Sun J. R., Brown J. M. (1989). Enhancement of the antitumor effect of flavone acetic acid by the bioreductive cytotoxic drug SR 4233 in a murine carcinoma.. Cancer Res.

[OCR_00308] Tocher J. H., Virk N. S., Edwards D. I. (1990). DNA damaging effects and voltammetric studies on the hypoxic cell toxin 3-amino-1,2,4-benzotriazine-1,4-dioxide, SR4233, as a function of pH.. Biochem Pharmacol.

[OCR_00314] Zeman E. M., Brown J. M., Lemmon M. J., Hirst V. K., Lee W. W. (1986). SR-4233: a new bioreductive agent with high selective toxicity for hypoxic mammalian cells.. Int J Radiat Oncol Biol Phys.

[OCR_00320] Zeman E. M., Brown J. M. (1989). Pre- and post-irradiation radiosensitization by SR 4233.. Int J Radiat Oncol Biol Phys.

